# **Retraction statement:** Sirtuin 6 contributes to migration and invasion of osteosarcoma cells via the ERK1/2/MMP9 pathway

**DOI:** 10.1002/2211-5463.13513

**Published:** 2022-11-15

https://doi.org/10.1002/2211-5463.12265

The above article published online on 01 July 2017 in Wiley Online Library (wileyonlinelibrary.com), has been retracted by agreement between the Editor‐in‐Chief, John Wiley and Sons Ltd and the authors. The retraction has been agreed following an investigation based on allegations raised by a third party, which revealed that some images in this article are inappropriate duplications of images from previously published articles [1, 2, 3, 4]. Thus, the editors consider the conclusions of this manuscript substantially compromised.
